# Serum 25(OH)D Levels Modify the Association between Triglyceride and IR: A Cross-Sectional Study

**DOI:** 10.1155/2022/5457087

**Published:** 2022-05-10

**Authors:** Rongpeng Gong, Xin Tang, Ziying Jiang, Gang Luo, Chaofan Dong, Xiuxia Han

**Affiliations:** ^1^Qinghai University, Xining, Qinghai 810016, China; ^2^Renal Department, Dezhou People's Hospital, Dezhou, Shandong Province 25300, China

## Abstract

**Background:**

Triglycerides and 25(OH)D had been reported as correlates of IR, but the results suggest substantial heterogeneity across races. In addition, little research reported on whether different 25(OH)D levels affect triglycerides and IR. Therefore, a similar study on the US population would be a great addition to the current one. This study investigated the association between triglycerides and IR at different 25(OH)D levels.

**Methods:**

A total of 19,926 participants were included, each containing specific indicators for the study project. IR was estimated as a HOMA-IR index ≥2.73. Four multivariate logistic regression models were developed to analyze the association between TG and IR and whether different 25(OH)D levels influenced this association. Smoothed fitting curves were plotted.

**Results:**

Triglyceride was significantly associated with IR (OR: 1.3, 95 CI %), while this association received different 25(OH)D levels (*P* for interaction <0.001). The effect value OR was 1.33 with the high levels, and its effect value OR was 1.28 with the low levels.

**Conclusion:**

This study demonstrates that triglyceride levels are significantly associated with insulin in the US adult population and can be used as a predictor of IR. This correlation was compromised at different 25 (OH)D levels, so future studies need to be explored in more ethnically diverse contexts.

## 1. Introduction

Insulin resistance (IR) is defined as reduced insulin sensitivity (IS) and refers to an increased amount of insulin needed to perform its metabolic actions [1]. According to previous research, IR is a hallmark of human obesity and is associated with the risk of developing diabetes, breast cancer, cardiovascular disease [2, 3], pancreatic cancer [2, 4], and liver cancer [5]. Nonalcoholic fatty liver disease (NAFLD) is a common cause of chronic liver disease and is closely associated with insulin resistance [6, 7]. Moreover, a recent study suggests that risk factors for insulin resistance include 25(OH)D deficiency, secondary hyperthyroidism, exercise intolerance, obesity, high triglyceride (TG) levels, and so on [8]. It can be deduced that 25(OH)D3 is a protective factor for IR, and TG is a risk factor for IR.

Vitamin D (25(OH)D), also known as cholecalciferol, is not really a vitamin but the precursor to the potent steroid hormone calcitriol (also named as 1,25-dihydroxy25(OH)D3 (1,25(OH)2 D3)), which has extensive roles through many tissues of the body. 25(OH)D was synthesized in human skin under the exposure to ultraviolet or through dietary uptake is well-adapted to the human body [9]. 25(OH)D3 deficiency may also be linked to autoimmune and infectious diseases such as osteoporosis [10], inflammatory bowel disease [11], glomerular disease, and chronic renal insufficiency [12]. In addition, 25(OH)D deficiency increases mortality from certain diseases such as systemic lupus erythematosus [13], liver cirrhosis [14], and diabetes [15]. Currently, there have been some studies showing that 25(OH)D promotes the synthesis and secretion of insulin by pancreatic *β*-cells [10, 16] and has a role in combating type I and II diabetes, and 25(OH)D3 deficiency is thought to be a significant factor in the development of type II diabetes.

Triacylglycerols (TG), fatty acyl ester derivatives of glycerol, are a class of neutral lipids that represent the most important storage form of energy for eukaryotic cells [17]. The primary function of TG is to serve as a vital energy substance for the body [18]. However, recent studies have shown that elevated TG levels can increase the severity of diseases, such as accelerating cardiovascular diseases and atherosclerosis [19], exacerbating gout symptoms [20], causing recurrent acute pancreatitis and persistent organ failure [21], and complicating dialysis patients with increased TG levels that also cause diabetes mellitus [22], coronary artery disease [23], male sexual dysfunction [24], nontraumatic fractures in middle-aged women [25], Alzheimer's disease [26], hypothyroidism [27], and one of the factors in the occurrence of diseases, especially diabetes mellitus. In addition, it has been demonstrated that increased muscle TG levels contribute to IR by attenuating insulin signaling [28].

It has been found that IR increases the risk of developing type II diabetes [29]. In recent years, type II diabetes (T2D) has become a global challenge with a tremendous economic burden for society and public health systems. More than 90% of patients with diabetes have type II diabetes and it is of great necessity to investigate the association between TG and IR at different 25(OH)D3 levels. It has been shown that TG is associated with IR [28]. However, there are not enough evidence to show whether 25(OH)D levels influence this association. With the present study, we sought to determine the effects of 25(OH)D levels and TG levels on IR using data from the 2009–2018 NHANES.

## 2. Methods

### 2.1. Data Sources

This study was conducted using a cross-sectional study of nine cycles (2001–2018) of data from the NHANES database (https://www.cdc.gov/nchs/nhanes/) of the National Center for Health. It is a study focusing on health screening and healthy eating in the United States. After applying its recommended population weights weighting, it applied a multistage stratified probability design to collect data. Its sample is representative of the overall sample of noninstitutionalized US citizens. These data included demographic data, dietary data, physical measurements, laboratory data, and questionnaire data. All NHANES-based studies were approved by the US National Health Statistics Research Ethics Review Board. Ethical approval and more detailed information can be found on the NHANES Ethics Review Committee's website (https://www.cdc.gov/nchs/nhanes/irba98.htm) [30].

### 2.2. Study Design and Participant Population

This study was a cross-sectional study and used data from the NHANES official website: https://www.cdc.gov/nchs/nhanes/NHANES. The target-independent variable was the level of serum triglyceride levels when the participants were tested. The dependent variable was IR, as defined by the participant's HOMA-IR index [31]. Less than 50 mmol/L was defined as the 25(OH)D deficient group, and ≥50 mmol/L was defined as the 25(OH)D level adequate group according to the internationally recommended definition of 25(OH)D levels.

Participants aged 20 years or older who completed an interview and examination at the Mobile Examination Center (MEC) from 2001 to 2018 were recruited for this study. Participants who do not meet the following criteria were excluded: (1) have data on fasting insulin, fasting glucose, and fasting triglycerides, (2) age ≥20 years, and (3) not taking medications that affect insulin, glucose metabolism, or lipid metabolism.

### 2.3. Data Collection

All data were collected by trained professionals. The data utilized in this study included demographics (age, gender, race, education, etc.), anthropometric measurements (height, waist circumference, weight, body mass index (BMI), etc.), health-related behaviors (smoking and alcohol consumption), and biochemical tests (HDL, ALT, AST, TG, etc.). All information is collected, and blood samples are collected in a mobile testing center (MEC) where basic information is collated immediately and serum samples are sent to the Laboratory Sciences Division of the National Center for Environmental Health, Centers for Disease Control and Prevention, and designated authorized institutions for analysis after scientific storage management.

#### 2.3.1. Measurement of 25(OH)D

Immediately after serum samples are taken at the MEC, they are stored frozen at −30°C. Samples for 25(OH)D measurement are then transported uniformly to the CDC Environmental Health Laboratory in Atlanta, Georgia. 25(OH)D levels (ng/ml) were defined as the sum of 25(OH)D3 and D2. Laboratory analysis was performed by ultrahigh performance liquid chromatography-tandem mass spectrometry (UHPLC-MS/MS) [32].

#### 2.3.2. Measurement of Triglycerides

The method for triglycerides was done based on Wahlefeld's method using lipases derived from microorganisms to facilitate the rapid and complete hydrolysis of triglycerides to glycerol and subsequent oxidation to dihydroxyacetone phosphate and hydrogen peroxide. In the presence of peroxidase, the peroxide reacts with 4-aminophenazone and 4-chlorophenol in a Trinder reaction to a colorimetric endpoint.

#### 2.3.3. HOMA-IR Index and Reduced Insulin Sensitivity

The HOMA-IR index is considered to be a good index for evaluating insulin sensitivity (PMID: 31958717). The formula for calculating HOMA-IR is: fasting blood glucose level (FPG, mmol/L) × fasting insulin level (FINS, *μ*U/mL)/22.5. In this study, based on previous studies, the HOMA-IR index in the US adult population was calculated as an IR index greater than or equal to 2.73 was defined as positive for IR in the US adult population, based on previous studies. Participants with a HOMA-IR index lower than 2.73 were defined as IR negative [33].

#### 2.3.4. Definition of Some Other Variables

Diabetes mellitus: fasting blood glucose was multiplied by 0.056 (rounded to retain three decimal places) to convert the unit from mg/dl to mmol/l [34]. Diabetes was diagnosed by fasting glucose ≥7.0 mmol/l, OGTT ≥11.1 mmol/l, physician diagnosis, self-reported, or taking diabetes medication.

Race: Mexican-American, other Hispanic, non-Hispanic white, non-Hispanic black, and other races.

Education: High school graduate, high school graduate, college graduate, or higher.

Smoking: smoked, quit smoking, and never smoked. Participants who smoked ≥100 cigarettes or more in total in the past and reported smoking on a few days or every day at the time of the interview were considered current smokers. Participants who smoked <100 cigarettes in the past but were not currently smoking were considered ex-smokers. Participants who smoked <100 cigarettes in the past were considered nonsmokers.

Alcohol consumption: drinking versus nondrinking. Heavy drinkers were defined as >1 drink/day for women and >2 drinks/day for men.

BMI: based on height and weight. Height was measured by the researchers using an electronic sports measuring device (Seca Ltd, Medical Scales and Measurement Systems, Birmingham, UK) with an accuracy of millimeters. Body weight was measured by researchers using a digital scale (Toledo Scale; Mettler-Toledo, LLC, Columbus, OH, USA), and after measurement, pounds were converted to kilograms. The formula for BMI is: BMI = weight (kg)/height (M^2^) [35].

### 2.4. Statistical Methods

All data were analyzed using R version 4.1.2, with continuous variables represented by detailed sample descriptions with a mean confidence interval of 95%. Categorical variables were represented by counts and weighted percentages. Skewed distributions were based on median and Q1–Q3. Normal distributions were described by median and standard deviation. Continuous variables were compared between groups using the Student *t*-test or Mann-Whitney *U* test based on the normality of the distribution, and within-group comparisons were made using Fisher's exact probability method. Covariates were selected based on potential confounders that may be associated with triglycerides and IR. Gender, age, race, smoking, BMI, obesity, and education were selected as covariates based on a combination of previous literature, international standards, and relevant clinical experience. Multiple interpolation was used to fill in the missing covariates with the aim of maximizing statistical power and minimizing bias. In addition, sensitivity analyses were conducted to see if the resulting complete data differed significantly from the original data. These studies showed that the data after multiple interpolation did not differ significantly from the original data and were not statistically significant (*p* > 0.05). Therefore, all results of our multivariate analysis were based on the data set after multiple interpolation according to Rubin's criterion. Four multivariate logistic regression models were developed to analyze the relationship with IR at different 25(OH)D levels, and smooth fitted curves were constructed. *p* < 0.05 (two-sided) was considered statistically significant.

## 3. Results

### 3.1. Description of the Basic Information of the Population

**Figure 1:**
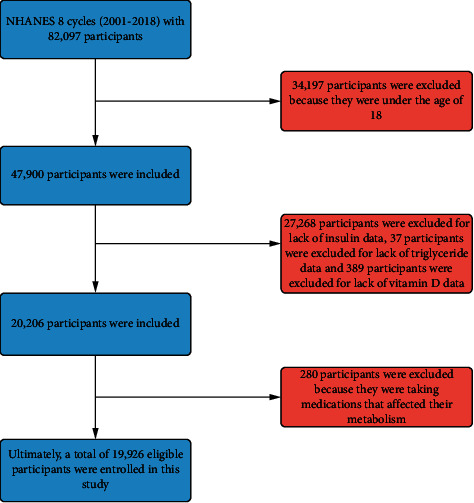
Flowchart of patient selection.

There were 19,926 participants in the 9 NHANES cycles (2001–2018; Figure 1). The basic information of the included participants is detailed in Table 1. The participants were grouped according to the occurrence of IR and divided into IR positive group and IR negative group. The mean age of all participants was 47.4 ± 19.2 years. The distribution of the two groups differed in gender, race, education, hypertension, diabetes, and alcohol consumption. BMI, waist circumference, TG, ALT, and AST were higher in the IR positive group than in the IR negative group. In contrast, HDL, TBIL, and 25(OH)D were higher in the IR negative group than in the IR positive group.

### 3.2. Univariate Regression Analysis

Univariate logistic regression showed the factors associated with IR in this study as shown in Table 2. Race, education, alcohol consumption, HDL, TBIL, ALB, and 25(OH)D were negatively associated with IR. In contrast, BMI, waist circumference, hypertension, diabetes, TG, ALT, AST, and BUN were positively associated with IR.

### 3.3. Multifactorial Regression Analysis

This study constructed four logistic regression models to analyze the independent association between triglyceride levels and IR. As shown in Table 3, the model-based effect values OR indicated a corresponding increase in the risk of IR for each unit increase in triglycerides. For example, in the unadjusted model (Model 1), the total effect value was 2.02 (1.94 to 2.09). Each 1 unit increase in triglycerides implies a 102% increase in the risk of developing IR. In the adjusted basic informatics model (Model 2), the effect value was OR: 2.07 (1.99 to 2.15). In the model adjusted for characteristic informatics (Model 3), the effect value was OR: 1.53 (1.46 to 1.59). In the fully adjusted model (Model 4), the OR and 95% CI were 1.3 and 1.25 to 1.36, respectively. The results suggest that triglycerides and IR were independently and negatively associated after controlling for potential confounders.

In addition, we also grouped according to 25(OH)D levels and observed whether triglycerides and IR received an effect of this association at different 25(OH)D levels (Table 3). The association between triglycerides and IR was strengthened within the high 25(OH)D subgroup (Model 1: 2.13 vs. 1.97, Model 2: 2.12 vs. 2.01, Model 3: 1.54 vs. 1.52, and Model 4: 1.33 vs. 1.28).

### 3.4. Curve-Fitting Analysis

**Figure 2:**
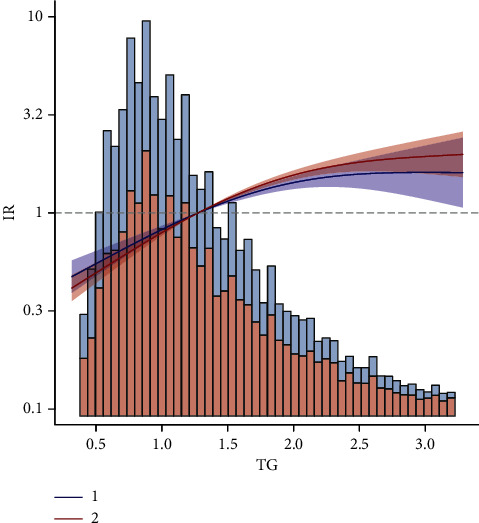
Association between TG and IR (total).

**Figure 3:**
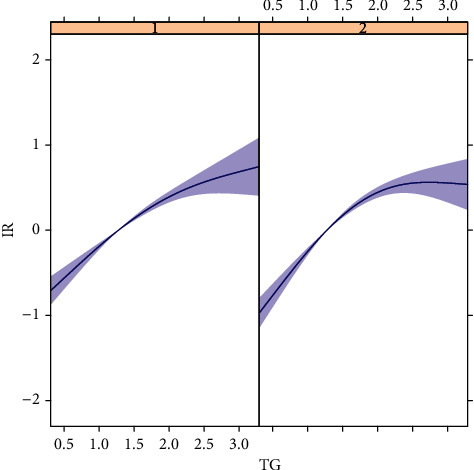
Association between TG and IR (individual).

The current study better explains the association between triglycerides and IR at different 25(OH)D levels. We plotted the curve fit. As shown in Figures 2 and 3, there is a difference in the association between different 25(OH)D levels and IR. Also, at higher triglycerides, higher 25(OH)D levels are more likely to be insulin resistant, which represents that high levels of triglycerides may weaken the protective effect of 25(OH)D on IR.

## 4. Discussion

To our knowledge, this study examined the association between triglyceride and IR using univariate logistic regression. It found that HDL, total bilirubin, albumin, and 25(OH)D may be protective factors for IR. In contrast, smoking, BMI and waist circumference above normal, hypertension, diabetes mellitus, triglycerides, glutathione, glutathione, and blood urea nitrogen may be risk factors for IR. In addition, men were found to have a higher probability of IR than women, in which the probability of IR varied significantly by race and the population's education level was negatively associated with the probability of IR. In the present study, we found that the odds of IR were lower in the drinking population than in the nondrinking population. However, this does not mean that we support alcohol drinking. More correlational studies need to evaluate the combined effects of alcohol consumption on humans.

The effect of confounding was explored by adjustment for a wide range of potentially confounding factors in regression models. In a fully adjusted model excluding other confounders (Model 4; Table 3), we found a strong correlation between TG and IR (OR: 1.3, 95% CI 1.25 to 1.36). Our results are largely consistent with those of other studies. For example, a cross-sectional study conducted by Boursier [36] in 2017 on an obese population (*n* = 498) found that triglycerides are an independent correlate of IR (OR: 3.0, 95% CI 2.0 to 4.5). In addition, Lin et al. [37] also found higher triglycerides in the Chinese population (*n* = 9764) with IR. In these studies, we found there are still some differences between their results and ours. The reasons for the differences may be due to the different populations selected for the studies (different races) and differences in the way the data were processed. Also, this study further found that the association between TG and IR differed significantly at different 25(OH)D3 levels, and the reason for the difference was that high levels of TG could disrupt the protective effect of 25(OH)D3 on IR.

Current mainstream studies report that 25(OH)D3 may improve IR by increasing insulin sensitivity through inhibition of inflammation [38–40]. 25(OH)D may reduce the extent of IR-related pathology by enhancing insulin signaling. There are various pathways through which 25(OH)D enhances insulin signaling, such as maintaining normal resting levels of ROS and Ca2+ [41], exerting autocrine and paracrine effects [42], and reducing oxidative stress [43]. It is thus clear that 25(OH)D may inhibit IR through multiple pathways.

On the other hand, some studies have shown that excess triglycerides in the body promote ectopic lipid accumulation in the liver, skeletal muscle, and heart triggering impairment of insulin signaling pathways and hence inducing IR [44, 45]. This is due to white adipocyte tissue (WAT) dysfunction caused by lipid accumulation. WAT dysfunction increases proinflammatory adipokines, and activation of oxidative stress and the renin-angiotensin-aldosterone system (RAAS) promotes IR [44, 46].

Notably, several studies have shown that high levels of TG lead to a decrease in 25(OH)D3 levels [47–50], suggesting that TG is negatively correlated with 25(OH)D3, which may be explained by the fact that cholecalciferol load is distributed in a larger volume, resulting in its release into the adipose tissue stores of 25(OH)D in circulation more slowly [50]. From these studies, we hypothesized that elevated TG levels would disrupt the protective effect of 25(OH)D3 on IR, leading to a stronger relationship between triglycerides and IR.

In addition, there is still some discussion about the link between insulin resistance and 25(OH)D. In today's world, the most prevalent chronic liver disease is chronic hepatitis due to nonalcoholic fatty liver disease. Most studies suggest that metabolic pathways such as 25(OH)D levels and insulin resistance may underlie nonalcoholic steatohepatitis. However, the conclusions of these studies are not absolute; for example, the findings of Yuval et al. [51] in 2016 suggest that 25(OH)D levels do not appear to be associated with NAFLD. Thus, despite preclinical evidence linking 25(OH)D to the pathogenesis of NAFLD, in humans, 25(OH)D deficiency does not appear to be associated with the severity of NAFLD. As Tarantino et al. [52] showed in 2019, this may be a critical point, as there is clear evidence that we are far from having a clear understanding of the underlying mechanisms of NAFLD.

We found shortcomings in the present study. For example, this study did not address some special populations, such as pregnant women and children. Whether the results apply to these special populations is currently unknown. More studies should be conducted in the future to demonstrate the applicability of the results to these specific populations. A 2014 study of 25(OH)D by Youselzadeh et al. [53] concluded that factors such as race and obesity were affected by 25(OH)D and 25(OH)D-binding proteins. At the same time, a 2017 study by Jassil et al. [54] found that non-Hispanic blacks had lower 25(OH)D, which more favourably demonstrated the link between 25(OH)D and 25(OH)D-binding proteins. In future research, we will also conduct research on 25(OH)D-binding proteins. However, there are clear advantages to this study. First, the sample size was large (19,926) and spanned a period of 18 years. Furthermore, the stratified fit curves were developed to better illustrate the association between triglycerides and IR. Moreover, we analyzed the association between triglycerides and IR using multiple regression, which allowed us to exclude further factors that were not strongly associated with IR. Only then did we finally conclude that high levels of triglycerides undermine the protective effect of 25(OH)D on IR.

## 5. Conclusion

We found an independent negative correlation between triglycerides and IR in the US population using a cross-sectional study. In addition, the association between TG and IR was enhanced under high-level 25(OH)D conditions when classified according to 25(OH)D levels. The study results may provide a reference for the analysis of factors in the pathogenesis of IR and suggest 25(OH)D levels and TG as markers of IR.

## Figures and Tables

**Table 1:** Baseline characteristics of the study participants.

Variables	Total (*n* = 19,926)	IR negative (*n* = 11,251)	IR positive (*n* = 8,675)	*P* value
Age, mean ± SD	47.4 ± 19.2	46.1 ± 19.5	49.1 ± 18.7	<0.001
Gender, *n* (%)				
Male	9,563 (48.0)	5,285 (47)	4,278 (49.3)	0.001
Female	10,363 (52.0)	5,966 (53)	4,397 (50.7)	
Race, *n* (%)				
Mexican American	3,578 (18.0)	1,745 (15.5)	1,833 (21.1)	<0.001
Other Hispanic	4,007 (20.1)	2,162 (19.2)	1,845 (21.3)	
Non-Hispanic white	8,981 (45.1)	5,449 (48.4)	3,532 (40.7)	
Non-Hispanic black	1,656 (8.3)	831 (7.4)	825 (9.5)	
Other races	1,704 (8.6)	1,064 (9.5)	640 (7.4)	
Education, *n* (%)				
Poorly educated	2,330 (11.7)	1,151 (10.2)	1,179 (13.6)	<0.001
Moderately educated	7,544 (37.9)	4,099 (36.4)	3,445 (39.7)	
Highly educated	10,052 (50.4)	6,001 (53.3)	4,051 (46.7)	
BMI, mean ± SD	28.6 ± 6.7	25.8 ± 4.9	32.2 ± 7.1	<0.001
Waist, mean ± SD	97.9 ± 16.2	90.9 ± 12.8	107.0 ± 15.6	<0.001
Hypertension, *n* (%)				
No	12,444 (62.5)	7,902 (70.2)	4,542 (52.4)	<0.001
Yes	7,482 (37.5)	3,349 (29.8)	4,133 (47.6)	
DM, *n* (%)				
No	16,537 (83.0)	10,394 (92.4)	6,143 (70.8)	<0.001
Yes	3,389 (17.0)	857 (7.6)	2,532 (29.2)	
Alcohol, *n* (%)				
No	15,810 (79.3)	8,547 (76)	7,263 (83.7)	<0.001
Yes	4,116 (20.7)	2,704 (24)	1,412 (16.3)	
Smoke, *n* (%)				
Never smoking	10,890 (54.7)	7,898 (70.2)	2,646 (30.5)	<0.001
Former smokers	4,942 (24.8)	2,813 (25)	2,134 (24.6)	
Current smokers	4,094 (20.5)	540 (4.7)	3,895 (44.9)	
HDL, median(IQR), mmol/L	1.3 (1.1, 1.6)	1.4 (1.2, 1.8)	1.2 (1.0, 1.4)	<0.001
TG, median (IQR), mmol/L	1.2 (0.8, 1.8)	1.0 (0.7, 1.4)	1.5 (1.0, 2.2)	<0.001
ALT, median (IQR), U/L	20.0 (16.0, 28.0)	19.0 (15.0, 25.0)	23.0 (17.0, 32.0)	<0.001
AST, median (IQR), U/L	23.0 (19.0, 27.0)	22.0 (19.0, 26.0)	23.0 (19.0, 28.0)	<0.001
TBIL, median(IQR), *μ*mol/L	12.0 (8.6, 15.4)	12.0 (10.3, 15.4)	12.0 (8.6, 13.7)	<0.001
BUN, median(IQR), mmol/L	4.3 (3.6, 5.4)	4.3 (3.2, 5.4)	4.3 (3.6, 5.7)	<0.001
ALB, median (IQR), g/L	42.0 (40.0, 45.0)	43.0 (41.0, 45.0)	42.0 (40.0, 44.0)	<0.001
25(OH)D, median (IQR), ng/ml	59.1 (43.3, 76.2)	62.2 (45.9, 80.1)	54.8 (40.0, 70.8)	<0.001

DM, diabetes mellitus; HDL, high-density lipoprotein; TG, triglyceride; ALT, alanine transaminase; AST, aspartate transaminase; TBIL, total bilirubin; BUN, blood urea nitrogen; ALB, albumin; and vitd, 25(OH)D.

**Table 2:** Univariate analysis for IR.

Variable	OR (95% CI)	*P* value
Age	1.01 (1.01 ∼ 1.01)	<0.001
Gender		
Male	1	
Female	0.91 (0.86 ∼ 0.96)	0.001
Race		
Mexican American	1	
Other Hispanic	0.81 (0.74 ∼ 0.89)	<0.001
Non-Hispanic white	0.62 (0.57 ∼ 0.67)	<0.001
Non-Hispanic black	0.95 (0.84 ∼ 1.06)	0.342
Other races	0.57 (0.51 ∼ 0.64)	<0.001
Education		
Poorly educated	1	
Moderately educated	0.82 (0.75 ∼ 0.9)	<0.001
Highly educated	0.66 (0.6 ∼ 0.72)	<0.001
BMI	1.22 (1.21 ∼ 1.23)	<0.001
Waist	1.09 (1.08 ∼ 1.09)	<0.001
Hypertension		
No	1	
Yes	2.15 (2.03 ∼ 2.28)	<0.001
DM		
No	1	
Yes	5 (4.6 ∼ 5.44)	<0.001
Alcohol		
No	1	
Yes	0.61 (0.57 ∼ 0.66)	<0.001
HDL	0.16 (0.15 ∼ 0.17)	<0.001
TG	2.02 (1.94 ∼ 2.09)	<0.001
ALT	1.02 (1.02 ∼ 1.03)	<0.001
AST	1 (1 ∼ 1.01)	<0.001
TBIL	0.96 (0.95 ∼ 0.96)	<0.001
BUN	1.04 (1.03 ∼ 1.05)	<0.001
ALB	0.94 (0.94 ∼ 0.95)	<0.001
25(OH)D	0.99 (0.99 ∼ 0.99)	<0.001

DM, diabetes mellitus; HDL, high-density lipoprotein; TG, triglyceride; ALT, alanine transaminase; AST, aspartate transaminase; TBIL, total bilirubin; BUN, blood urea nitrogen; and ALB, albumin.

**Table 3:** Multiple logistic regression analysis of the association between TG and IR.

Variable	Model 1		Model 2		Model 3		Model 4	
	OR (95% CI)	*P* value	OR (95% CI)	*P* value	OR (95% CI)	*P* value	OR (95% CI)	*P* value
TG	2.02 (1.94∼2.09)	<0.001	2.07 (1.99∼2.15)	<0.001	1.53 (1.46∼1.59)	<0.001	1.3 (1.25∼1.36)	<0.001
25(OH)D group								
<50 nmol/L	1.97 (1.84∼2.1)	<0.001	2.01 (1.87∼2.15)	<0.001	1.52 (1.41∼1.63)	<0.001	1.28 (1.19∼1.37)	<0.001
≥50 nmol/L	2.13 (2.03∼2.23)	<0.001	2.12 (2.02∼2.23)	<0.001	1.54 (1.47∼1.62)	<0.001	1.33 (1.26∼1.4)	<0.001

Model 1: nonadjusted; Model 2: adjusted age, gender, and race; Model 3: adjusted age, gender, race, and education, BMI, waist, hypertension, DM, and alcohol; Model 4: adjusted age, gender, race, education, BMI, waist, hypertension, DM, alcohol, ALT, AST, TBIL, BUN, ALB, and HDL.

## Data Availability

All data were downloaded from NHANES' official website: https://www.cdc.gov/nchs/nhanes/. NHANES was a large multipurpose cross-sectional survey that provided comprehensive data on various aspects of nutrition and health.

## Abbreviations

BMI: Body mass index
FBG: Fasting blood glucose
VitD3: Vitamin D3
Hb-A1c: Glycosylated hemoglobin
TC: Total cholesterol
ALT: Alanine aminotransferase
BUN: Blood urea nitrogen
WC: Waist
TG: Triglyceride
CI: Confidence interval
OR: Odds ratio
HUA: Hyperuricemia.
